# No effects of a long-term physical activity intervention on executive functioning among adolescents

**DOI:** 10.1177/2050312119880734

**Published:** 2019-10-04

**Authors:** Douglas Sjöwall, Lisa B Thorell, Mirko Mandic, Maria Westerståhl

**Affiliations:** 1 The Division of Psychology, Department of Clinical Neuroscience, Karolinska Institutet, Stockholm, Sweden; 2 Habilitation and Health, Stockholm County Council, Stockholm, Sweden; 3 Division of Clinical Physiology, Department of Laboratory Medicine, Karolinska Institutet, Stockholm, Sweden; 4 Department of Clinical Physiology, Karolinska University Hospital, Stockholm, Sweden

**Keywords:** Cognition, mental health, psychiatry, physical exercise, school-based intervention, sports medicine

## Abstract

**Objectives:**

We investigated whether a school-based physical activity intervention would lead to improvements in working memory, inhibition and cognitive flexibility in adolescents aged 13–15 years.

**Methods:**

The adolescents at the active school (*n* = 108) participated in an intervention that included increased physical activity for 20 min/day, focused on aerobic activity with low cognitive demands for an entire school year. The adolescents at the control school (*n* = 59) received no extra physical activity. At the beginning (baseline) and end (follow-up) of the school year, the participants performed tests of executive function (working memory, inhibition and cognitive flexibility) and performed tests of physical fitness and health.

**Results:**

There was no change in executive functioning at follow-up when comparing the schools. However, only 46% complied with the intervention. When non-compliers were excluded from the analyses, the results remained the same, except for a small but significant increase in working memory for the active school as compared to the control school.

**Conclusion:**

These results indicate that compliance with the intervention was low and that aerobic exercise with low cognitive load does not produce improvements in executive functioning.

## Background

There is a strong trend toward introducing more physical activity in the school curriculum to improve academic performance. This is based on the belief that physical activity has a positive effect on executive functioning, which in turn is important for school performance.^1^ The bases for how these effects could be produced are multifold^2^ and include neurobiological mechanisms such as brain-derived neurotrophic factor (BDNF) which in turn could affect plasticity.^3^ Positive effects of physical activity on executive functioning have been reported, but effect sizes are often relatively small and there is great heterogeneity between studies.^2^ Furthermore, a recent review concluded that there is a lack of controlled studies targeting adolescents.^4^ Adolescence represents a critical period in life during which behavioral patterns are established and previous studies have shown that the proportion of active individuals declines significantly in adolescence.^5^ Thus, interventions aiming to increase physical activity in this age group might be of especially high importance. In this study, we therefore included adolescents aged 13–15 years with the aim to investigate the effects of increased physical activity (20 min a day for an entire school year) on what have been suggested to be the three major components of executive functioning: inhibition, cognitive flexibility and working memory.^6^

### Executive functioning and physical activity

Working memory is the ability to retain and manipulate information over a short period of time,^7^ and it is regarded as one of the most important cognitive functions for academic performance.^8^ A recent meta-analysis of controlled trial^2^ found significant improvements on working memory, although with a small effect size (*d* = 0.14). This meta-analysis did not include separate analyses for different types of physical activity interventions, but recognized the need to address whether some types of physical activity could be more beneficial than others. The results from previous studies on preadolescents (ages 7–12 years) show that exercise with a high cognitive load (e.g. complex motor skill exercise) appears to have a stronger effect^9–12^ compared to high-intensity aerobic exercises with low cognitive load.^9,13^ A similar conclusion was made in the meta-analyses referred to above.^2,4^

Another major aspect of executive functioning is inhibition. It has been defined as the ability to purposefully stop a dominant response to perform a deliberate action.^14^ This ability is, for example, important for ignoring distracting stimuli in the classroom. Based on controlled trials,^2^ a significant impact of physical activity on inhibition has been found, although with a small effect size (*d* = 0.26). A recent study of 12- to 15-year-olds found beneficial effects on neurophysiological indices of inhibitory control, but not on accuracy, using an 8-week-long intervention including both aerobic and coordination exercises.^15^ Another study found larger effects on inhibition for overweight compared to non-overweight children using a cognitively challenging physical activity intervention.^16^

A third major aspect of executive functioning is cognitive flexibility, sometimes referred to as shifting. It is defined as the ability to shift between tasks or mental sets.^6^ This ability is required when task demands change and when it is useful to view a given situation from a new perspective. Very few controlled studies have included cognitive flexibility, and the significant but small effect (*d* = 0.11) presented in the meta-analyses should therefore be interpreted with caution.^2^ Results of previous studies are even more mixed compared to those presented above for working memory and inhibition. In one previous study, academic lessons including physical activity and cognitive demands did not enhance cognitive flexibility in preadolescents.^17^ However, in a study of third graders, cognitive flexibility improved more when including high intensity and high cognitive load compared to a control condition with low intensity and low cognitive load.^18^ In another study of this age group, including mainly high-intensity physical activity for 9 months, there was a significant effect on cognitive flexibility.^19^ To our knowledge, only one controlled study has investigated adolescents and this study showed a significant increase in cognitive flexibility after a 6-month physical activity intervention.^20^

### Aim

The research presented above suggests that the effect of physical activity on executive functioning is still unclear. Furthermore, findings from recent meta-analyses within this area of research included mostly children aged 6–12, and we therefore do not know whether increased physical activity is beneficial for executive functioning in adolescence. The aim of this study was therefore to investigate whether an aerobic physical activity intervention has a significant effect on working memory, inhibition or cognitive flexibility in adolescents aged 13–15 years.

## Methods

### Procedure and participants

This was a controlled study with one active school and one control school being assessed with tests of physical performance, health and executive functioning at the beginning (baseline) and at the end of a school year (follow-up 9 months later). A nearby school was chosen as the control school to include participants with similar socio-economical background. All students in seventh to ninth grades at both the active school and the control school were eligible for participation. In total, 108 adolescents (65 girls and 43 boys) at the active school and 59 adolescents (39 girls and 20 boys) at the control school where included in the study. The proportion of girls and boys who participated in the study did not differ between the two schools (*p* = 0.45).

### The intervention

The intervention was initiated and designed by the active school. Based on previous research, the assumption was that executive functions would be affected by increased physical activity. During one full school year, 20 min of daily, outdoor physical activity (circuit training and dance moves) was included in the curriculum and scheduled in conjunction with the lunch break. Two teachers were responsible for the daily physical activity and participation was mandatory. At follow-up, compliance was checked by asking the participants to assess the statement “This is what I think about having physical activity every day” with alternatives “I like it and I do it every day,” “I don’t like it but I do it anyway,” “I don’t like it and I don’t participate” and “I don’t know because . . .” Those who chose either the first or the second alternative were categorized as compliers. No extra physical activity was performed in the control school.

### Ethical approval and informed consent

The study was reviewed and approved by the local ethics committee in Stockholm. Written informed consent was obtained from the adolescents’ legal guardians as well as from the adolescents themselves.

### Instruments

#### Anthropometrics and health

Body weight (kg) and height (m) were measured in light clothing and without shoes. Body mass index (BMI) was calculated (kg/m^2^) and the participants were categorized according to guidelines^21^ as having normal, overweight and obesity.

Systolic and diastolic blood pressure (mmHg) was measured in triplicates, in supine position, in the left arm after at least 10 min rest, using an automatic oscillometric blood pressure device (Omron Model M6 Comfort, Omron Healthcare Ltd, UK). In cases where the blood pressure according to guidelines^22^ was elevated, the adolescents (*n* = 2) were asked to make an appointment with the school nurse.

#### Physical capacity

An adjustable handheld dynamometer (Jamar Hydraulic Hand Dynamometer, Patterson Medical, UK) was used to obtain handgrip strength measurements. The participants were positioned in an upright sitting position, with shoulders in neutral rotation at their side and 90° elbow flexion. The highest handgrip strength after three consecutive attempts, separated by brief pauses (30 s), was registered.

#### Motor skills

The protocol used to evaluate the adolescents’ physical performance and motor skills was modified from that used in a previous study.^23^ It included 10 different movements and tasks that were visually graded by three observers on a 4-point scale ranging from “major shortcomings” to “highly satisfactory.” Each level of the scale had a written description to support the ocular observations. Examples of skills tested were skipping, jumping, rolling, bouncing and throwing balls.

#### Executive functioning

The tests measuring executive functioning were chosen from two of the most well-known test batteries: the fifth edition of the Wechsler Intelligence Scale for Children (WISC-V)^24^ and the Delis–Kaplan Executive Function System (D-KEFS).^25^

Verbal working memory was measured using the backward and sequencing conditions from the Digit Span Subtest from WISC-V.^24^ The administrator reads a series of numbers and the task is to repeat the numbers in a backward order (i.e. backward condition) or in the correct number order (i.e. sequencing condition). Number of points, with 1 point being awarded for each correct trial, was used as a measure of working memory.

Inhibition was measured using the interference trial from the Color Word Interference Test from D-KEFS.^25^ Participants are shown rows of words printed in conflicting colors (e.g. the word green printed in red color) and are asked to inhibit reading the words and instead name the color in which the words are printed. The number of seconds needed to complete the trial was used as a measure of inhibition.

Cognitive flexibility was measured using the shifting trial from the Color Word Interference Test from D-KEFS.^25^ Participants are instructed to switch between naming the dissonant print color and reading the word. Completion time was used as a measure of set shifting.

### Data analyses

Outliers were detected using the outlier labeling rule^26^ and adjusted to the upper value (third quartile + (2.2 × the interquartile range)) or lower value (first quartile + (2.2 × the interquartile range)) for each group. First, the two schools were compared for all variables at baseline using *t* tests (dimensional variables) or chi-square tests (categorical variables). To investigate the effect of the intervention, participants’ functioning at follow-up was compared using analyses of covariance (ANCOVAs) with group (active school vs control school) as the between-subjects factor and baseline performance as the covariate. Effect sizes were calculated using partial eta-square (η^2^) in accordance with recommendations,^27^ 0.01 was considered a small effect, 0.06 a medium-sized effect and 0.14 a large effect.

Next, *t* tests were carried out to investigate differences between compliers and non-compliers at the active school and all ANCOVAs were re-run with non-compliers excluded. Delta scores (baseline to follow-up) were created and correlations were used to investigate associations between change in physical/motor functioning and change in executive functioning.

## Results

Descriptive data are shown in Table 1. A large majority of the adolescents were of normal weight at both the active (90%) and the control schools (81%) and the proportion of adolescents with normal weight versus overweight/obesity did not differ between the schools (χ^2^ = 2.31, *p* = 0.13). There were no group differences regarding parental education (χ^2^s < 1.9, *p*s > 0.40), sex (χ^2^ = 0.57, *p* = 0.45) or age (*t* = 1.40, *p* = 0.16). Adolescents at the control school had significantly higher values at baseline for heart rate (*t* = 3.07, *p* < 0.01), diastolic blood pressure (*t* = 3.66, *p* < 0.001) and systolic blood pressure (*t* = 3.26, *p* < 0.01). However, the blood pressure was within the normal range for all adolescents except two. Moreover, adolescents at the control school had significantly higher values for backward digit recall (*t* = 2.12, *p* < 0.05), but group differences were not significant for the other physical and executive variables (all *t*s < 0.88, *p*s > 0.53).

**Table 1.** Comparison of all participants at the active and control school post-intervention while controlling for pre-intervention measures.

	Intervention group (*n* = 108)		Control group (*n* = 59)		ANCOVA (η^2^)
	Baseline M (SD)	Follow-up M (SD)	Baseline M (SD)	Follow-up M (SD)	*F*
Physical measures					
BMI (kg/m^2^)	20.0 (2.9)	20.0 (3.0)	20.0 (3.5)	20.5 (3.0)	1.33 (0.01)
Heart rate (beats/min)	76 (14)	75 (15)	82 (13)	77 (13)	0.28 (0.00)
Hand grip right (kg)	28 (8)	31 (6)	29 (11)	33 (11)	2.88 (0.02)
Hand grip left (kg)	26 (8)	29 (6)	26 (11)	31 (10)	1.53 (0.01)
Motor functions (units/points)	2.7 (0.5)	2.7 (1.1)	2.6 (0.6)	2.9 (0.5)	1.33 (0.01)
Diastolic blood pressure (mmHg)	69 (7)	70 (6)	73 (6)	71 (7)	0.79 (0.01)
Systolic blood pressure (mmHg)	103 (10)	107 (8)	109 (12)	106 (11)	12.00 (0.08)***
Executive functions					
Inhibition					
Stroop task—Time(s)	60 (12)	53 (11)	58 (12)	51 (10)	1.74 (0.01)
Stroop task—Errors (number)	2.6 (2.4)	1.5 (1.7)	2.5 (2.5)	1.0 (1.2)	3.64 (0.02)
Cognitive flexibility					
Stroop—Time (s)	67 (13)	58 (12)	66 (11)	57 (9)	0.25 (0.00)
Stroop—Errors (number)	3.04 (3.14)	1.57 (1.60)	3.00 (2.54)	1.33 (1.66)	0.75 (0.01)
Working memory					
Digit span—Backward (points)	9.3 (2.3)	10.1 (2.1)	10.2 (2.20)	10.0 (2.2)	3.29 (0.03)
Digit span—Sequential (points)	9.2 (2.0)	9.3 (2.1)	9.4 (2.1)	9.1 (2.1)	1.75 (0.01)

BMI: body mass index; ANCOVA: analysis of covariance.

Means (M) and standard deviations (SD) for all physical measures, executive functions included in the study and *F* values for ANCOVAs.

*** *p* < 0.001.

As shown in Table 1, there were no significant group differences after the intervention for the executive functions or physical variables, except that the systolic blood pressure increased at the active school and decreased at the control school.

With regard to compliance, the results showed that only 46% of the adolescents at the active school participated in the daily physical activity (i.e. classified as compliers). There were no significant differences at baseline between compliers and non-compliers regarding age, parental occupation, parental education, BMI, handgrip, motor functions, heart rate, blood pressure or sex (all *p*s > 0.08). Regarding executive functioning, non-compliers performed significantly better at baseline on the inhibition task and the backward digit recall task (all *t*s > 2.20, *p*s < 0.05). However, group differences were non-significant (all *p*s > 0.53) for the other measures of executive functioning.

To further investigate the effects of the intervention, we compared compliers at the active school with participants at the control school. The results (see Table 2) showed no significant group differences at follow-up, except for systolic blood pressure and backward digit recall, which measures working memory. For systolic blood pressure, the effect was the same as that found for the whole sample (i.e. an increase at the active school and a decrease at the control school). For working memory, the adolescents at the control school performed better than the compliers at the active school before the intervention, but the groups did not differ after the intervention.

**Table 2.** Comparing the compliers at the active school and all participants at the control school post-intervention while controlling for pre-intervention measures.

	Intervention group (compliers) (*n* = 43)		Control group (*n* = 59)		ANCOVA (η^2^)
	Baseline M (SD)	Follow-up M (SD)	Baseline M (SD)	Follow-up M (SD)	*F*
Physical measures					
BMI (kg/m^2^)	19.7 (2.6)	19.5 (2.7)	20.0 (3.5)	20.5 (3.0)	2.43 (0.03)
Heart rate (beats/min)	77 (15)	77 (13)	82 (13)	77 (13)	0.38 (0.01)
Hand grip right (kg)	29 (9.3)	31 (8)	29 (11)	33 (10)	0.24 (0.02)
Hand grip left (kg)	27 (10)	30 (7)	26 (11)	31 (10)	0.37 (0.01)
Motor functions (units/points)	2.7 (0.43)	2.8 (1.1)	2.6 (0.6)	2.9 (0.5)	0.34 (0.00)
Diastolic blood pressure (mmHg)	69 (6)	70 (5)	73 (6)	71 (7)	0.09 (0.00)
Systolic blood pressure (mmHg)	104 (11)	108 (8)	109 (12)	106 (11)	9.34 (0.09)**
Executive functions					
Inhibition					
Stroop task—Time(s)	63 (13)	54 (10)	58 (12)	51 (10)	0.50 (0.01)
Stroop task—Errors (number)	3.1 (2.5)	1.6 (1.6)	2.5 (2.5)	1.0 (1.2)	3.30 (0.04)
Cognitive flexibility					
Stroop—Time (s)	67 (11)	58 (12)	66 (11)	57 (9)	0.06 (0.00)
Stroop—Errors (number)	2.79 (2.09)	1.71 (1.73)	3.00 (2.54)	1.33 (1.66)	1.75 (0.02)
Working memory					
Digit span—Backward (points)	8.49 (2.02)	9.9 (1.9)	10.2 (2.20)	10.0 (2.2)	4.80 (0.06)*
Digit span—Sequential (points)	8.95 (2.02)	9.1 (2.5)	9.4 (2.1)	9.1 (2.1)	0.98 (0.01)

BMI: body mass index; ANCOVA: analysis of covariance.

Means (M) and standard deviations (SD) for all physical measures, executive functions included in the study and *F* values for ANCOVAs.

* *p* < 0.05; **p < 0.01.

There were no significant associations between change in handgrip strength and change in executive functioning (all *p*s > 0.15) among compliers at the active school. Nor were there any significant associations between change in motor functioning and change in executive functioning (all *p*s > 0.33).

## Discussion

The main finding of this study was that there were no effects of aerobic physical activity on executive functioning. Moreover, even though the active school initiated this intervention, there were difficulties implementing it, as only about half of the adolescents participated in the physical activity on a daily basis. These results raise concerns about the strong trend toward introducing physical activity interventions to improve school performance. Below, we discuss the results in detail and thereafter conclude with a broader discussion to identify future directions within this field of research.

The main objective of the active school was to improve executive functioning. However, this study showed no effects on executive functioning of adding 20 min per day of aerobic physical activity. Although this is in line with studies on preadolescents and adults,^4^ very few controlled studies have shown this in adolescence. Moreover, because many previous interventions have included a mix of physical and cognitive demands, this study enables an evaluation of a physical activity intervention focused on aerobic exercise with low cognitive demand.

One of the few beneficial effects of the intervention was that compliers at the active school increased their working memory capacity significantly more compared to participants at the control school. However, this effect was only found for one of the two verbal working memory measures. There is a possibility that this could be explained as an effect of regression toward the mean, given that the adolescents at the control school performed better at baseline, whereas the groups did not differ at follow-up. Even if this is a real effect, the medium effect size should be interpreted in relation to the effort made and the time invested in the intervention (i.e. 20 min a day for a full school year, which amounts to a total of about 60 h of extra physical exercise). Thus, a critical question is whether this effect should be considered large enough or whether there are other, more effective intervention efforts?

With regard to our finding that the blood pressure of the adolescents at the active school increased, while it decreased at the control school, it should be noted that changes were small. Thus, the effect is most likely due to differences in location for the testing between baseline and follow-up at the control school. It is possible that the higher blood pressure at baseline compared to follow-up at the control school was caused by a noisier room where the blood pressure was taken at baseline. Normally, blood pressure does not decrease, but increases by ~1–2 mmHg per year during adolescence.^28^

To better understand the lack of significant effects, it is important to take a closer look at this particular intervention. Previous studies have suggested that it is not necessarily the change in cardiovascular fitness or anthropometrical characteristics that determines improvements in executive functioning. There are studies showing effects on fitness without effects on executive functioning,^13^ and studies showing improvements in executive functioning without any association to changes in fitness.^16^ Moreover, there are studies indicating that it is improvements in motor skills, rather than changes in fitness, that produce improvements in executive functioning.^9^ Regarding motor functioning, this study was not designed to train such abilities and the lack of significant effects are therefore not surprising. In addition, changes in motor functions was not associated with changes in executive functioning. This gives further support to the suggestion that it may be cognitively demanding activities that drive improvements in executive functioning rather than aerobic exercise. If the present intervention had produced changes in the physical variables without improvements in executive functioning, this would be even stronger evidence for the conclusion that changes in physical activity do not produce improvements in executive functioning.

One important finding of this study was the limited compliance, with 54% not participating regularly in the intervention. Non-compliance was not related to differences in background factors (i.e. sex, parental education), nor did compliers and non-compliers differ significantly with regard to the physical measures. Thus, it was not the adolescents with high physical fitness at baseline who chose to participate because they already liked sports. Nor was it the adolescents with the lowest values who felt the need to participate. Unexpectedly, however, there was some indication that non-compliers performed better on executive tests at baseline. We cannot find evidence from any previous studies to explain this effect.

### Limitations, conclusions and future directions

The large percentage of non-compliers could have contributed to the absence of an intervention effect. However, data were re-analyzed using only those who participated actively, something seldom done in previous studies. The sample size was substantially reduced when we excluded non-compliers, which could limit the ability to detect small effects. However, because the results were in line with those of the whole group and because effect sizes were small, there is no reason to believe that the non-existing effects of the intervention could be due to lack of power.

The participants in this study were healthier than the average boy and girl in Sweden with regard to BMI and blood pressure values.^22^ It could be that adolescents with good physical fitness do not benefit as much as those with poor physical fitness. Regarding executive functioning, participants performed within the range of what is considered normal for inhibition and cognitive flexibility.^25^ Thus, there should have been room for improvement in executive functioning.

The type of physical intervention used in this study was not effective, but this does not mean that physical activity would not, under any circumstances, produce gains in executive functioning. It could be that more than 20 min are needed. Furthermore, it could also be that physical activity has an effect on executive functioning, but that the 2 h of training the control school also offered each week was sufficient.

## Conclusion

Conclusively, the results of this study showed that the participants’ compliance with daily physical activity within a school-based intervention was low and that aerobic exercise with low cognitive load did not produce improvements in executive functioning.

At this point, it is not clear why some physical activity interventions produce positive improvements in executive functioning and others do not. Future studies should compare high intensity with high cognitive load with low physical activity but the same cognitive load. This would address the question of whether it is the cognitive load or the combination of high-intensity physical activity and cognitive load that is necessary.
